# Microarray Analysis and Detection of MicroRNAs Associated with Chronic Thromboembolic Pulmonary Hypertension

**DOI:** 10.1155/2017/8529796

**Published:** 2017-08-21

**Authors:** Ran Miao, Ying Wang, Jun Wan, Dong Leng, Juanni Gong, Jifeng Li, Yunxia Zhang, Wenyi Pang, Zhenguo Zhai, Yuanhua Yang

**Affiliations:** ^1^Department of Clinical Laboratory, Beijing Chao-Yang Hospital, Capital Medical University, Beijing 100020, China; ^2^Key Laboratory of Respiratory and Pulmonary Circulation Disorders, Institute of Respiratory Medicine, Beijing 100020, China; ^3^Department of Pulmonary and Critical Care Medicine, China-Japan Friendship Hospital, Beijing 100029, China; ^4^Department of Respiratory and Critical Care Medicine, Beijing Chao-Yang Hospital, Capital Medical University, Beijing 100020, China

## Abstract

The aim of this study was to understand the importance of chronic thromboembolic pulmonary hypertension- (CTEPH-) associated microRNAs (miRNAs). miRNAs differentially expressed in CTEPH samples compared with control samples were identified, and the target genes were predicted. The target genes of the key differentially expressed miRNAs were analyzed, and functional enrichment analyses were carried out. Finally, the miRNAs were detected using RT-PCR. Among the downregulated miRNAs, MiR-3148 regulated the most target genes and was significantly enriched in pathways in cancer, glioma, and ErbB signaling pathway. Furthermore, the number of target genes coregulated by miR-3148 and other miRNAs was the most. AR (androgen receptor), a target gene of hsa-miR-3148, was enriched in pathways in cancer. PRKCA (Protein Kinase C Alpha), also a target gene of hsa-miR-3148, was enriched in 15 of 16 KEGG pathways, such as pathways in cancer, glioma, and ErbB signaling pathway. In addition, the RT-PCR results showed that the expression of hsa-miR-3148 in CTEPH samples was significantly lower than that in control samples (*P* < 0.01). MiR-3148 may play an important role in the development of CTEPH. The key mechanisms for this miRNA may be hsa-miR-3148-AR-pathways in cancer or hsa-miR-3148-PRKCA-pathways in cancer/glioma/ErbB signaling pathway.

## 1. Introduction

Chronic thromboembolic pulmonary hypertension (CTEPH), a complication of acute pulmonary embolism, is characterized by the persistence of a thromboembolic obstruction of the pulmonary arteries by organized tissue and the presence of variable small vessel arteriopathy [1]. In 2015 ESC (European Society of Cardiology)/ERS (European Respiratory Society) Guidelines for the diagnosis and treatment of pulmonary hypertension (PH), CTEPH is classified as the fourth types of PH [2]. It is reported that CTEPH has a cumulative incidence of 0.1–9.1% within the first 2 years after a symptomatic pulmonary embolism event [3]. Risk factors for CTEPH include circulating antiphospholipid antibodies or lupus anticoagulant, increased factor VIII, non-O blood groups, and chronic inflammatory diseases [4]. The survival of CTEPH patients is poor in the absence of specific surgical or medical treatment [4]. Therefore, there is an urgent need for effective treatments for CTEPH.

With the rapid development of bioinformatics, high-throughput microarray data analysis plays an important role in the study of the molecular mechanism of disease. Pathways enriched by differentially expressed genes and interactions between genes can provide theoretical basis for the mechanisms of disease occurrence and development. MicroRNAs (miRNA), small noncoding RNAs, are differentially expressed in many cardiovascular diseases, including pulmonary hypertension (PH) [5]. A previous study indicated that levels of miR-125a were increased in the lung tissues of hypoxic animals that developed PH [6]. Courboulin et al. suggested that miR-204 plays a significant role in decreasing proliferation, vascular remodeling, and pulmonary artery blood pressure in PH [7]. Furthermore, the fibrinogen alpha gene regulated by miR-759 is associated with a susceptibility to CTEPH [8]. Wang et al. suggested that miRNA let-7d may play important roles in the pathogenesis of CTEPH [5]. Therefore, miRNAs may be important biological molecules to understand the mechanisms of CTEPH. However, the miRNAs associated with CTEPH have not been fully characterized.

To understand the miRNAs associated with CTEPH, we carried out microarray analysis and detection of miRNAs. Firstly, miRNAs differentially expressed in CTEPH samples compared control samples were identified, and the target genes of these differentially expressed miRNAs were predicted. Then, the target genes of the key differentially expressed miRNA were analyzed, and functional enrichment analyses were carried out. Finally, the miRNAs were detected using RT-PCR.

## 2. Materials and Methods

### 2.1. miRNAs Expression Profile Data

Peripheral blood of CTEPH patients (4 samples in CTEPH group) in Beijing Chao-Yang Hospital, Capital Medical University, and healthy volunteer (5 samples in control group) with routine physical examination in physical examination center from March to April 2016 were collected. This study was approved by the Ethics Committee of Beijing Chao-Yang Hospital, Capital Medical University. The requirement to obtain informed written consent was waived. The information about the patients was shown in Table 1.

Total RNAs of the samples were extracted following the manufacturer's protocol by the RNAprep Pure Blood Kit (Tiangen Biotech Co., Ltd., Beijing, China), and then RNA was purified with mirVana™ miRNA Isolation Kit (AM1561). Quantification was performed by using spectrophotometer or Qubit, and quality control was carried out by using agarose gel electrophoresis or Agilent 2100. Total RNA was labeled by poly(A) polymerase addition using the Genisphere FlashTag HSR kit following the instructions of the manufacturer instructions (Genisphere, Hatfield, PA). RNA was hybridized to the Affymetrix miRNA array. Chips were washed and stained by using Affymetrix® GeneChip® Command Console® Software (AGCC). After scanning, fluorescent scan images were saved in  .DAT files with AGCC. A total of 9 human blood samples (4 samples: 160039k_1, 160039k_2, 160039k_3, and 160039L_1 in the CTEPH group; 5 samples: 160039J_6, 160039J_7, 160039J_8, 160039J_9, and 160039J_10 in the control group) were included in the Affymetrix miRNA chip.

### 2.2. Screening for Differentially Expressed miRNAs

Data preprocessing including robust multiarray averaging (RMA) normalization, discrimination of probe signal, and integration of probe set signal was performed by using Expression Console package provided by Affymetrix. SAM (significance analysis of microarray) R software package [9] with *q* values ≤ 0.05 and |log⁡2FC| > 1.0 was used for the identification of differentially expressed miRNAs.

### 2.3. Prediction Analysis for Target Genes of the Differentially Expressed miRNAs

Combined with the results of the miRWalk, Microt4, miRanda, mirbridge, miRDB, miRMap, miRNAMap, Pictar2, PITA, RNA22, RNAhybrid, and Targetscan databases, prediction analysis to determine the target genes of the differentially expressed miRNAswas carried out using miRWalk2.0 (http://zmf.umm.uni-heidelberg.de/apps/zmf/mirwalk2/) [10, 11]. Prediction results greater than six were regarded as being the result of regulation of a target gene by the miRNA, and differentially expressed miRNA-target gene pairs were obtained.

### 2.4. Functional Enrichment Analysis for Differentially Expressed miRNAs

The number of target genes regulated by differentially expressed miRNAs was counted, and KEGG pathway enrichment analysis was performed for the top 5 differentially expressed miRNAs by using clusterProfiler in R package [12]. *P* < 0.01 was set as the threshold values.

### 2.5. Target Genes Coregulated by Differentially Expressed miRNAs Analysis

The coregulation network of two miRNAs was constructed using the coregulated target genes of the two miRNAs. The networks for these microRNAs were constructed using Cytoscape software [13].

### 2.6. The Network Construction for Target Genes Regulated by Differentially Expressed miRNAs

The target genes regulated by more differentially expressed miRNA were regarded as key target genes. The top 100 target genes regulated by more miRNAs were obtained and the network was constructed with these target genes and miRNAs.

### 2.7. Functional Enrichment Analysis for Target Genes of Key miRNAs

GO [14] and Kyoto Encyclopedia of Genes and Genomes (KEGG) [15] pathway enrichment analysis were carried out for the target genes regulated by key miRNAs using the DAVID (Version 6.8, https://david-d.ncifcrf.gov/) online tool (classification stringency = medium) [16]. *P* < 0.05 was set as the threshold values.

### 2.8. Detection of miRNAs Using RT-PCR

A total of 11 RNA samples (CTEPH group: K-1, K-2, K-3, K-4, and SN6 and control group: J6, J7, J8, J9, J10, and MN-N2) were used for the detection of miRNAs. Based on previous studies and our experience, we measured the expression of hsa-miR-3148. The primers for the miRNA are shown in Table 2.

Poly(A) was added to the 3′ end of the miRNA as follows: firstly, 1 *μ*l 10x EPAP Reaction Buffer, 1 *μ*l 25 mM MnCl_2_, 1 *μ*l 10 mM ATP, 6.5 *μ*l total RNA, and 0.5 *μ*l* Escherichia coli* poly(A) polymerase were added to a precooled RNase-free reaction tube with a total volume of 10 *μ*l. The prepared reaction solution was gently mixed using transferpettor, and the reaction was performed at 37°C for 60 min after transient centrifugation. The obtained solution was used for a subsequent experiment or transiently preserved at −20°C (long-term storage at −80°C).

The reverse transcription reaction mixture was prepared as follows: firstly, 3 *μ*l RT-Primer (10 *μ*M) and 1 *μ*l dNTP Mixture (10 mM each) were added to the 10 *μ*l prepared reaction solution and then RNase-free water was added up to 20 *μ*l. The denaturation reaction was performed at 65°C for 5 min. The mixture was then precooled on ice. Then, 4 *μ*l 5x PrimeScript II Buffer, 0.5 *μ*l (20 U) RNase Inhibitor (40 U/*μ*l), 1 *μ*l (200 U) PrimeScript II RTase (200 U/*μ*l), and 0.5 *μ*l RNase-free dH_2_O were added to 14 *μ*l of the above denaturation reaction solution, and the solution was mixed using a transferpettor. Then, after transient centrifugation, the reverse transcription reaction was performed at 42°C for 60 min and 95°C for 5 min and then cooled on ice [17].

Then, the qPCR reaction solution was prepared according to the following components: 10 *μ*l SYBR Premix EX Taq (2x), 1 *μ*l forward primer 10 *μ*M, 1 *μ*l reverse primer 10 *μ*M, and 8 *μ*l cDNA. The qPCR reaction was performed using the following steps: 50°C for 3 min, 40 cycles of 95°C for 3 min, 95 for 10 s, and 60°C for 30 s. Finally melt curve analysis was carried out in 60–95°C using increments of 0.5°C per 10 s.

All results are presented as the mean ± SEM and presented in tables. SPSS22.0 was used for the statistical analyses, and GraphPad Prism 5 (GraphPad Software, San Diego, CA) was used for mapping. Values of *P* < 0.05 and *P* < 0.01 were set as a significant difference and an extremely significant difference.

## 3. Results

### 3.1. Screening of Differentially Expressed miRNA

**Figure 1:**
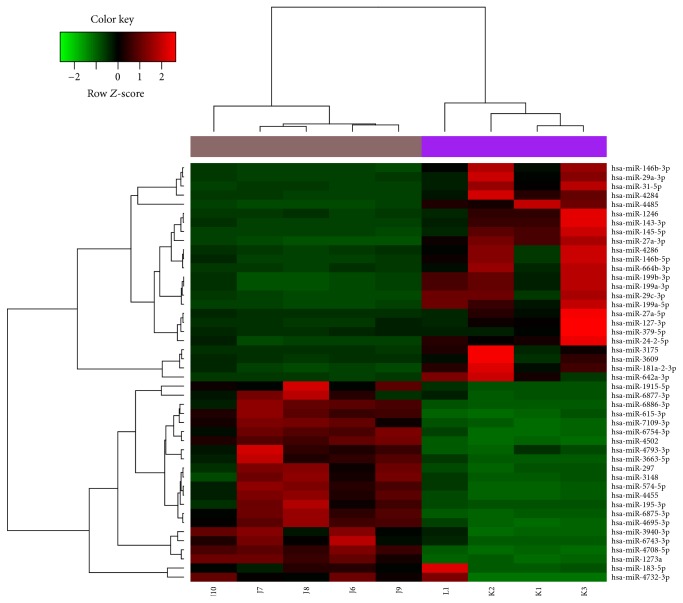
Heat map of the differentially expressed miRNA. The green represents lower expression levels, and the red represents higher expression levels.

A total of 46 (24 upregulated and 22 downregulated) differentially expressed miRNAs were obtained from comparing the CTEPH group compared with the control group. The heat map of these differentially expressed miRNAs is shown in Figure 1.

### 3.2. Target Gene of Differentially Expressed miRNA Prediction Analysis

A total of 34386 target gene pairs were obtained from upregulated miRNAs and 16751 from downregulated miRNAs. The top 10 results for the number of target genes regulated by differentially expressed miRNAs are shown in Table 3. Of the miRNAs, miR-3148 regulated the most target genes.

### 3.3. Functional Enrichment Analysis for Differentially Expressed miRNAs

**Figure 2:**
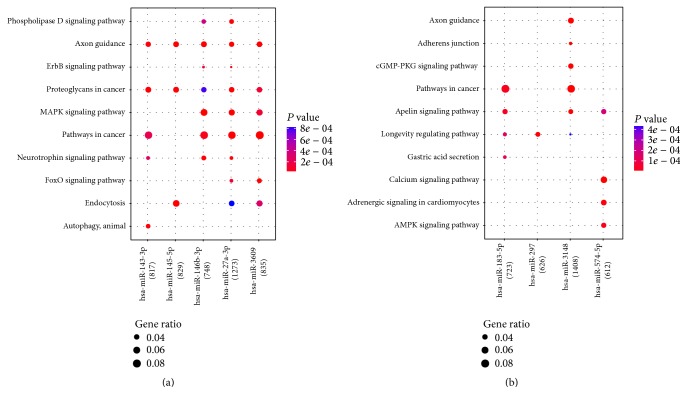
Functional enrichment analysis for differentially expressed miRNAs. Node size: gene ration; node color: the *P* value.

As shown in Figure 2, the top 5 upregulated miRNAs were mainly enriched in pathways in cancer and axon guidance, and the top 5 downregulated miRNAs were mainly enriched in pathways in cancer and apelin signaling pathway. Among them, miR-3148 was significantly enriched in pathways in cancer and axon guidance.

### 3.4. Target Genes Coregulated by Differentially Expressed miRNAs Analysis

**Figure 3:**
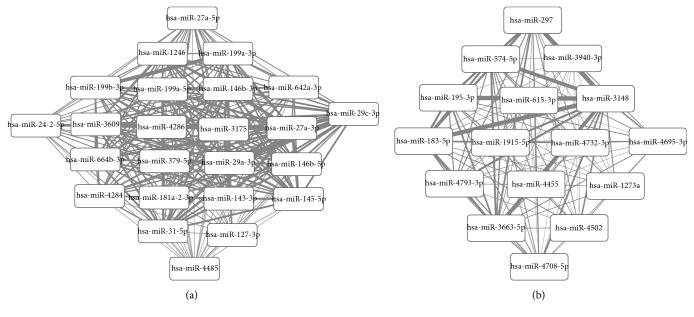
The coregulated networks for upregulated (a) and downregulated (b) differentially expressed miRNAs. The thickness of the line represents the number of target genes coregulated by two miRNAs.

The coregulated networks for upregulated and downregulated differentially expressed miRNAs were shown in Figure 3. The number of coregulated genes (top 10) was shown in Table 4. It showed that the number of target genes coregulated by miR-3148 and other miRNAs was the most.

### 3.5. The Network Construction for Target Genes Regulated by Differentially Expressed miRNAs

**Figure 4:**
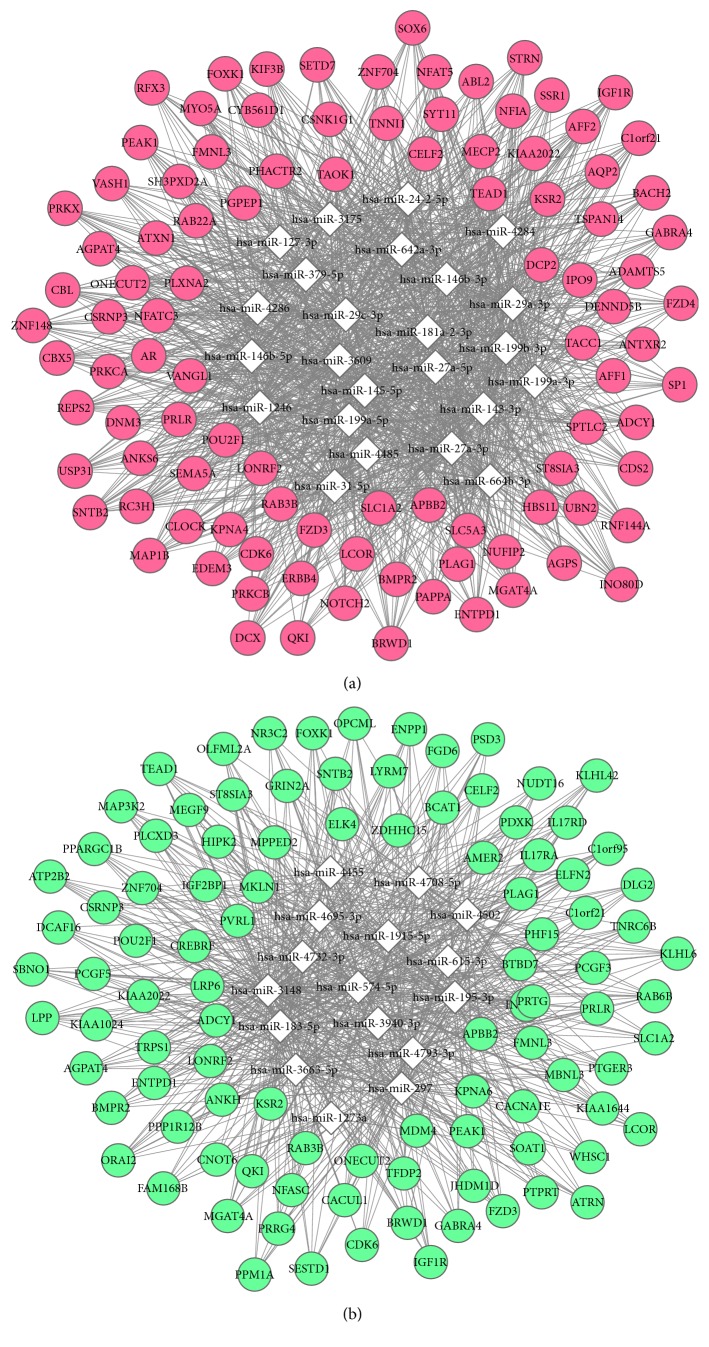
The miRNA-Target network for the upregulated (a) and downregulated (b) differentially expressed miRNAs. (a) Pink represents target genes; white represents upregulated differentially expressed miRNAs; (b) green represents target genes; white represents downregulated differentially expressed miRNAs.

We constructed the miRNA-Target network for the upregulated and downregulated differentially expressed miRNAs, respectively (Figure 4). ONECUT2 (One Cut Homeobox 2), RC3H1 (Ring Finger and CCCH-Type Domains 1), and SLC1A2 (Solute Carrier Family 1 Member 2) were regulated by 19 upregulated miRNAs; ONECUT2 and RAB6B (Member RAS Oncogene Family) were regulated by 11 downregulated miRNAs.

### 3.6. Functional Enrichment Analysis of the Target Genes of the Key miRNAs

The target genes regulated by upregulated differentially expressed miRNAs were mainly enriched in 21 GO terms and 16 KEGG pathways, and the target genes regulated by downregulated differentially expressed miRNAs were mainly enriched in 45 GO terms and calcium signaling pathway. Among them, the top 5 results were shown in Table 5. For example, AR (androgen receptor), a target gene of hsa-miR-3148, was enriched in pathways in cancer. PRKCA (Protein Kinase C Alpha), also a target gene of hsa-miR-3148, was enriched in 15 of 16 KEGG pathways, such as pathways in cancer, glioma, and ErbB signaling pathway.

### 3.7. Detection of miRNAs Using RT-PCR

**Figure 5:**
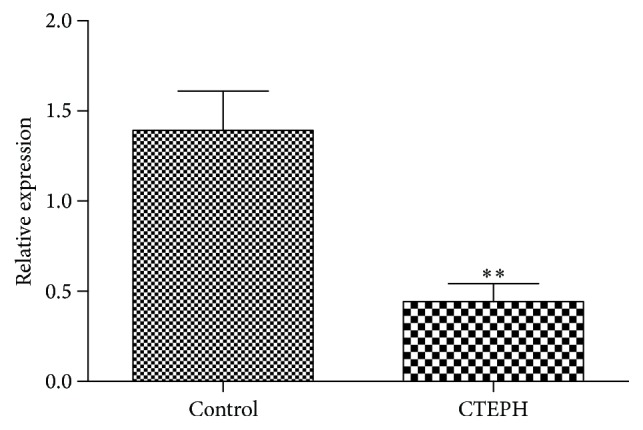
The expression of hsa-miR-3148 in CTEPH samples compared with that in control samples (“*∗∗*” represents *P* < 0.01).

As shown in Figure 5, the expression of hsa-miR-3148 in CTEPH samples was significantly lower than that of the control samples (*P* < 0.01).

## 4. Discussion

CTEPH is the fourth types of PH, and the roles of miRNAs in several diseases progression such as PH are becoming increasingly evident [5]. In the present study, we carried out microarray analysis and detection of miRNAs to understand the key miRNAs associated with CTEPH. The results showed that miR-3148 regulated the most target genes and was significantly enriched in pathways in cancer, glioma, and ErbB signaling pathway. Furthermore, the number of target genes coregulated by miR-3148 and other miRNAs was the most. AR (androgen receptor), a target gene of hsa-miR-3148, was enriched in pathways in cancer. PRKCA (Protein Kinase C Alpha), also a target gene of hsa-miR-3148, was enriched in 15 of 16 KEGG pathways, such as pathways in cancer, glioma, and ErbB signaling pathway. In addition, the RT-PCR results showed that the expression of hsa-miR-3148 in CTEPH samples was significantly lower than that in control samples (*P* < 0.01).

It has been reported that miRNA-3148 modulates the differential gene expression of the SLE- (systemic lupus erythematosus-) associated TLR7 (toll-like receptor 7) variant [18], and TLR7 mediates relaxation of airways through nitric oxide production [19]. In our present study, miR-3148 was demonstrated to be an important miRNA for CEPTH by bioinformatics analysis and RT-PCR. Therefore, although not too much previous studies reported the roles of miRNA-3148 in CEPTH, we inferred that miR-3148 may play important roles in CTEPH according to the present study.

Furthermore, AR, one target gene of hsa-miR-3148, was enriched in pathways involved in cancer. PRKCA, also a target gene of hsa-miR-3148, was enriched in pathways in cancer, glioma, and ErbB signaling pathway. The hsa-miR-3148 was significantly enriched in pathways in cancer, glioma, and ErbB signaling pathway. Previous studies have reported that androgens play a critical role in cardiovascular disease [20] and are associated with pulmonary arterial hypertension [21], and AR had been identified in the right and left ventricles [22]. The changes in membrane translocation and protein expression of cPKC*α*, *β*I, *β*II, and nPKC*δ* are involved in the development of hypoxia-induced rat pulmonary hypertension [23]. An organized thrombus in major pulmonary arteries is typically in association with other diseases, such as lung cancer [24]. There is a very high incidence of symptomatic venous thromboembolisms for patients with glioma [25]. Grant et al. indicated that modulation of ErbB signaling pathway could lead to increased cell apoptosis and loss of clonogenic survival [26], and cell proliferation was related to pulmonary hypertension [27, 28]. Although no previous studies have suggested direct associations between genes, including AR and PRKCA or pathways in cancer, gliomas, ErbB signaling pathway, and CTEPH, they led to our hypothesis that AR, PRKCA, and pathways in cancer, gliomas, and ErbB signaling pathway are associated with CTEPH. Combined with the results of the present study, we suggest that hsa-miR-3148 may play roles in CTEPH via hsa-miR-3148-AR-pathways in cancer or hsa-miR-3148-PRKCA-pathways in cancer/glioma/ErbB signaling pathway.

In conclusion, we suggest that hsa-miR-3148-AR-pathways in cancer or hsa-miR-3148-PRKCA-pathways in cancer/glioma/ErbB signaling pathway may be the key mechanisms in CTEPH. However, there are limitations in our study, such as the relatively small sample size; hence, further studies are needed.

## Figures and Tables

**Table 1:** The baseline characteristics of the 9 samples.

	Sex	Collection date	Age	BMI (kg/m^2^)	Family history of blood clots	Smoking	Long periods of inactivity	Other CTEPH risk factors
*CTEPH group*								
160039K-1	Male	March 2016	41	25.99	No	15 years, quit smoking for 3 year	No	No
160039K-2	Male	March 2016	67	23.66	No	40 years, quit smoking for 4 year	No	No
160039K-3	Female	March 2016	53	27.99	No	No	No	Unilateral lower extremity edema, a year before onset
160039L-1	Female	March 2016	71	18.96	No	No	No	Varicosity
*Control group*								
160039J-6	Male	April 2016	50	/	No	/	No	No
160039J-7	Male	April 2016	56	/	No	/	No	No
160039J-8	Female	April 2016	71	/	No	/	No	No
160039J-9	Female	April 2016	64	/	No	/	No	No
160039J-10	Male	April 2016	50	/	No	/	No	No

For smoking, we did not investigate this information for control group, but there was no correction between smoking and CTEPH according to previous studies. For BMI, we did not investigate this information for control group. 160039K-1, 160039K-2, 160039K-3, 160039L-1, 160039J-6, 160039J-7, 160039J-8, 160039J-3, and 160039J-10 were chip number.

**Table 2:** Sequences of miRNA primers.

Primer name	Primer sequence (5′-3′)
hsa-miR-3148-F	TGGAAAAAACTGGTGTGTGCTT
Universal downstream primer	GCTGTCAACGATACGCTACCTA
U6-F	CTCGCTTCGGCAGCACA
U6-R	AACGCTTCACGAATTTGCGT

**Table 3:** The top 10 results for the number of target genes regulated by differentially expressed miRNAs.

num	up_miRNA	num	down_miRNA
3211	hsa-miR-27a-3p	3679	hsa-miR-3148
2220	hsa-miR-143-3p	1756	hsa-miR-183-5p
2073	hsa-miR-145-5p	1700	hsa-miR-3663-5p
2072	hsa-miR-3609	1639	hsa-miR-574-5p
2066	hsa-miR-146b-3p	1636	hsa-miR-297
1953	hsa-miR-29a-3p	1443	hsa-miR-195-3p
1945	hsa-miR-31-5p	786	hsa-miR-1915-5p
1927	hsa-miR-29c-3p	666	hsa-miR-4793-3p
1820	hsa-miR-3175	533	hsa-miR-4708-5p
1802	hsa-miR-146b-5p	525	hsa-miR-4732-3p

**Table 4:** The number of coregulated genes (top 10).

	mir1	mir2	num
Upregulated	hsa-miR-29a-3p	hsa-miR-29c-3p	1818
	hsa-miR-199a-3p	hsa-miR-199b-3p	1248
	hsa-miR-3609	hsa-miR-27a-3p	838
	hsa-miR-143-3p	hsa-miR-27a-3p	797
	hsa-miR-145-5p	hsa-miR-27a-3p	792
	hsa-miR-29a-3p	hsa-miR-27a-3p	744
	hsa-miR-31-5p	hsa-miR-27a-3p	737
	hsa-miR-29c-3p	hsa-miR-27a-3p	726
	hsa-miR-27a-3p	hsa-miR-146b-5p	714
	hsa-miR-27a-3p	hsa-miR-146b-3p	702

Downregulated	hsa-miR-297	hsa-miR-3148	839
	hsa-miR-195-3p	hsa-miR-3148	836
	hsa-miR-183-5p	hsa-miR-3148	820
	hsa-miR-3148	hsa-miR-574-5p	678
	hsa-miR-3148	hsa-miR-3663-5p	657
	hsa-miR-297	hsa-miR-183-5p	410
	hsa-miR-195-3p	hsa-miR-297	406
	hsa-miR-4793-3p	hsa-miR-3148	372
	hsa-miR-195-3p	hsa-miR-183-5p	370
	hsa-miR-183-5p	hsa-miR-3663-5p	362

**Table 5:** Functional enrichment analysis of the target genes of the key miRNAs.

Term	Description	Count	*P* value
*Upregulated*			
GO:0060736	Prostate gland growth	3	8.49*E* − 04
GO:0006366	Transcription from RNA polymerase II promoter	10	2.30*E* − 03
GO:0045893	Positive regulation of transcription, DNA-templated	10	2.36*E* − 03
GO:0060749	Mammary gland alveolus development	3	3.99*E* − 03
GO:0018105	Peptidyl-serine phosphorylation	5	5.33*E* − 03
hsa05205	Proteoglycans in cancer	7	1.05*E* − 03
hsa04310	Wnt signaling pathway	6	1.24*E* − 03
hsa04012	ErbB signaling pathway	5	1.66*E* − 03
hsa05200	Pathways in cancer	9	1.86*E* − 03
hsa04916	Melanogenesis	5	2.77*E* − 03
*Downregulated*			
GO:0010557	Positive regulation of macromolecule biosynthetic process	10	5.24*E* − 03
GO:0006820	Anion transport	5	5.78*E* − 03
GO:0048666	Neuron development	7	7.04*E* − 03
GO:0031328	Positive regulation of cellular biosynthetic process	10	7.05*E* − 03
GO:0030278	Regulation of ossification	4	7.06*E* − 03
hsa04020	Calcium signaling pathway	5	2.59*E* − 03

Term represents the identification number of GO-BP or KEGG pathway. Description represents the name of the GO-BP or KEGG pathway. Counts represent the number of genes enriched in the GO-BP or KEGG pathway.

## Additional Points

*Highlights*. (1) Microarray analysis and detection of significant miRNA were performed. (2) MiR-3148 may play important roles in CTEPH. (3) The pathways in cancer, glioma, and ErbB signaling pathway may be vital for CTEPH.
